# Research progress of health care in Yemeni children during the war: review

**DOI:** 10.1017/S1463423622000421

**Published:** 2022-09-12

**Authors:** Yahya Ali Gaber, Rukaih Al-Sanabani, Dhekra Amin Annuzaili, Abdullah Al-danakh, Li Chun Ling

**Affiliations:** 1 Department of Maternal, Child and Adolescent Health, School of Public Health, Guangxi Medical University, Shuang-yong Road No. 22, Nanning, 530021, Guangxi, China; 2 School of Public Health, Jinan University, Guangzhou, China; 3 Pediatrician and Senior Public Health Consultant, MD, DCH, DIC, ECD, MPH. Ministry of Health & Population, Yemen; 4 Department of Urology, First Affiliated Hospital of Dalian Medical University, Dalian 116021, China

**Keywords:** children, diseases, health conditions, organizations, Yemen Crisis

## Abstract

**Background:**

Yemen crisis, which has been going on for more than six years, represents one of the most gruesome human plights in the modern history, especially children.

**Objectives:**

This research aimed to present a comprehensive view of Yemeni children’s studies during the ongoing war period, to come up with a comprehensive base that concerns humanitarians, researchers, decision-makers, and general public at large about the reality of the predicament of Yemeni child.

**Method:**

We searched databases and identified 373 articles, of which 68 were included in this review. Review of literature between 2014 and 2020 is taken from academic sources, multilateral organizations, donors, and governmental and non-governmental organizations. The data are analyzed by date and governorates.

**Results:**

We chose 68 articles and divided them according to the diseases and health conditions as follows: infectious diseases (15 studies), non-infectious diseases (10 studies), blood-related diseases (7 studies), oral and dental problems (12 studies), accidents and injuries (2 studies), health system (16 studies), family and community (6 studies). Moreover, the studies were divided geographically as follows: 7 studies that were almost comprehensive for all governorates; additional studies were conducted for Amanat Al Asimah (21 studies), Taiz (12 studies), Aden and Al Hudaydah (7 studies for each), Dhamar and Ibb (6 studies for each), Abyan and Lahij (2 study for each). As for Al Bayda, Marib, Sana’a, and Socotra, each of them had one study.

**Conclusion:**

Our assessment revealed that the ongoing Yemen crisis is underrated and largely neglected. The studies conducted so far do match the ground reality both in terms of inclusiveness and numbers.

## Background

Yemen is a low-income country that has a huge political, economic, structural, and health sector vulnerabilities, including widespread food insecurity, energy shortages, and water scarcity (Qirbi and Ismail, 2017). In 2015, it was reported that 35% of Yemen’s population lived below the national poverty line, particularly when the war was internationalized by the international coalition in late March 2015 (Boley, Kent *et al*., 2017; El Bcheraoui, Jumaan *et al*., 2018). Prior to 1 April 2021, almost 4 million people were internally displaced, out of which 79% account for women and children (UNHCR, 2021). Along with the waves of displacement, it is critical to precisely identify the health risks and problems that children encounter in their new environments, which has not been done in Yemen (Bosson, Williams *et al*., 2022). All of this contributed to a serious humanitarian catastrophe affecting Yemenis, particularly the most vulnerable populations (children) who were adversely impacted by food, health, and education deprivation. Most children have spent the majority of their lives in war zones, either directly or indirectly exposed to an armed conflict through harm and influence. The number of children affected by the war is not exact due to the geography of the widespread armed conflict; even in areas where there is no armed conflict, the collapse of health systems, food shortages, and high prices in exchange for scarce resources, a lack of employment, and wage cuts in the private and public sectors resulted in the United Nations Children’s Fund (UNICEF) announcing in February 2019 that at least 24.1 million people (80% of the population) needed humanitarian assistance, including 12.3 million children (El Bcheraoui, Jumaan *et al*., 2018).

Child health refers to the overall state of the physical, mental, intellectual, social, and emotional well-being of a child. Healthy children live in families, communities, and environments that enable them to realize their full developmental potential (National Research and Institute of 2004). UNICEF reports that Yemeni children continue to be killed and maimed during the conflict. Simultaneously, the damage and closure of schools and hospitals have disrupted access to basic education and health services. According to the organization’s February 2021 analysis, nearly 2.3 million children under the age of five in Yemen are predicted to suffer from acute malnutrition, with 400 000 suffering from severe conditions that will result in death, if not treated. Numerous factors, driven by war, economic decline and now exacerbated by the second wave of COVID-19, have compounded the dire situation for Yemen’s children (WHO, 2021).

The ongoing war has resulted in aggravating the health problems across the country and causing malnutrition in children (Burki, 2016). Several studies have published their findings based on the last national population survey that was conducted in 2013. Despite the significant changes in the population, the data collection has become extremely difficult, since the war divided the country into rebel-controlled and government-controlled regions (Oakford, 2019). With this challenge, there is an urgent need for research into the availability and quality of child and maternal health services, as well as adaptive responses during armed conflict (Obel, Martin *et al*., 2021).

Numerous studies conducted across the nations of Burundi, Nigeria, Eritrea, and Cote d’Ivoire have concluded that conflict has a detrimental effect on the healthy state of children and enforces chronic malnutrition among them (Acharya, Luke *et al*., 2020). Similarly, the effects of the conflict in Afghanistan, Iraq, and the Syrian Arab Republic accounted for more than 76% of all war-related deaths in 2016, including children (Dupuy and Rustad, 2018). Due to studies limitation, it is still difficult to determine the impact of war on children’s growth and health in Yemen, so, in this study, we present a comprehensive survey of the issues related to childhood in all of its aspects, categorizing them by geography. Then to draw attention to areas that did not receive appropriate research attention, as well as conflict areas that did not receive a proportion of studies proportional to the hazards facing children. Ultimately, we make the necessary recommendations to address these concerns in a war-torn region, implementing evidence-based decisions and allocating resources appropriately.

## Methods

We searched PubMed, Springer, and Google Scholar databases, and the official websites of World Health Organization (WHO) (www.who.int) and UNICEF (www.unicef.org). Yemen, children, war, health, childhood, and health care were among the most searched keywords. We conducted a literature research on child health care from 2014 to 2020, focusing exclusively on English-language sources. The initial literature search identified 772 articles, out of which 373 were selected for additional screening. The final shortlist contained 68 articles.

## Result and discussion

**Figure 1.**
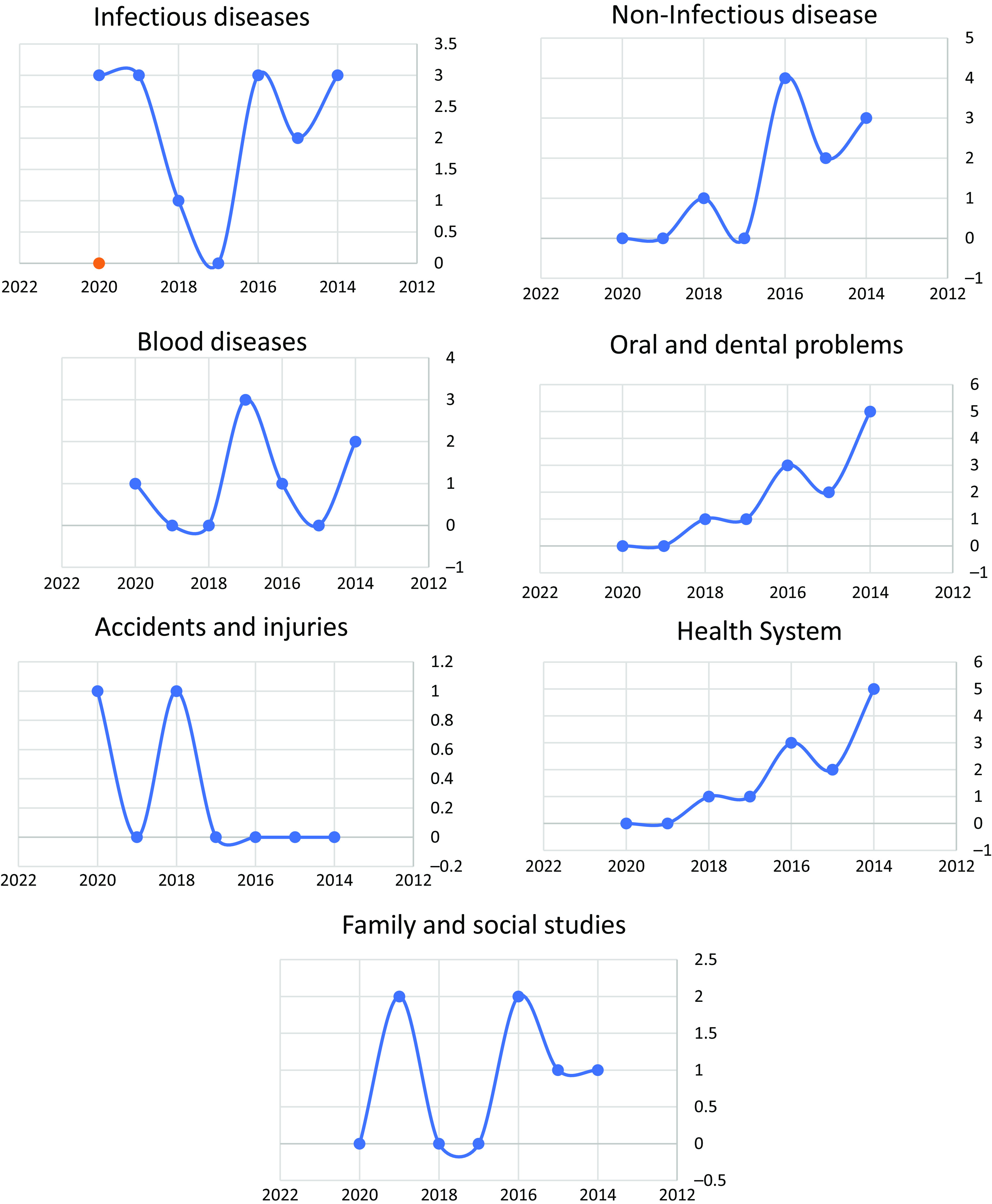
Shows the movement of researches according to topics during the war years from 2014 to 2020 (infectious diseases, non-infectious diseases, blood-related diseases, injuries and accidents, oral and dental problems, health system, family and society studies).

**Figure 2.**
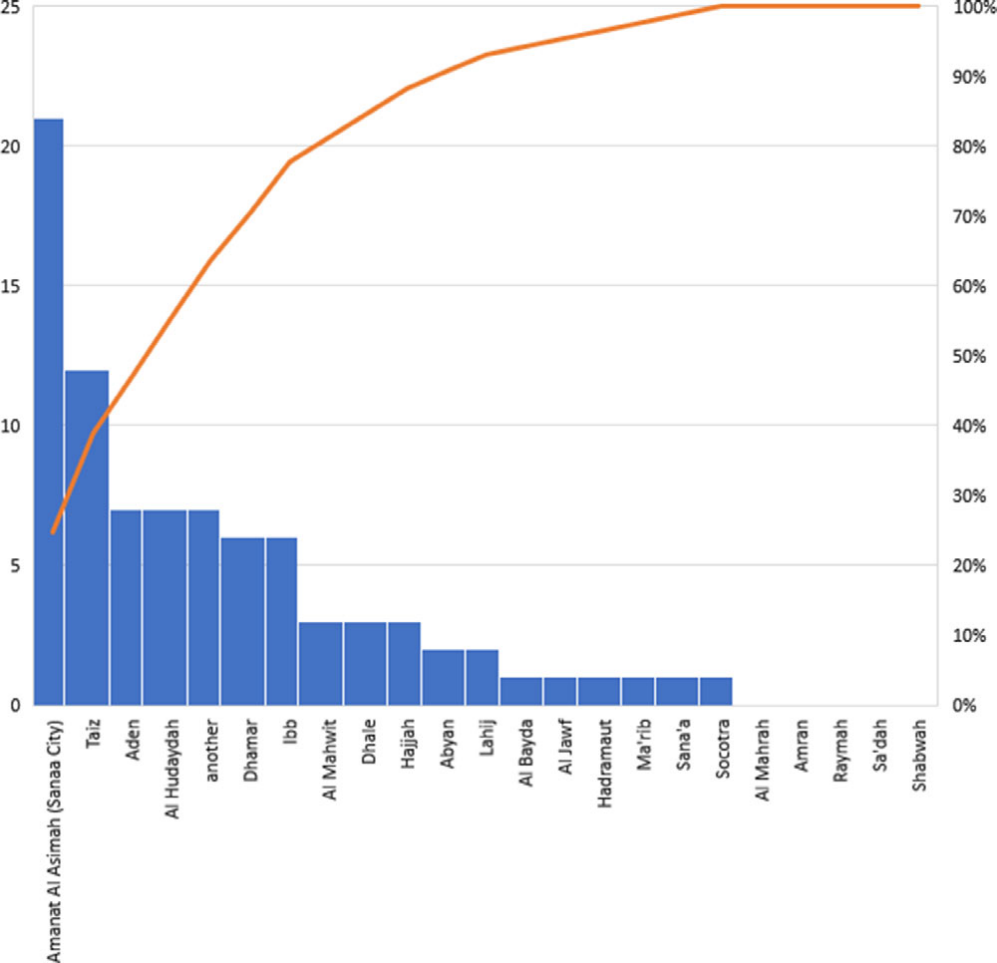
Showing the distribution of studies about children at the governorate level from 2014 to 2020.

This study summarizes the most significant findings from studies conducted on children between 2014 and 2020, and they are structured as follows: infectious diseases, non-infectious diseases, blood-related diseases, injuries and accidents, oral and dental problems, health system, family and society studies, and the relationship between these studies show variability based on time and years as illustrated in Figure 1. The detailed summary of the presented and selected studies is drawn in Table 1. The research and the extent to which they were dispersed and distributed at the governorate level resulted in a gradient that was unsuitable for population distribution or exacerbation of health problems, with certain governorates having no studies at all. The majority of studies (21 studies) were conducted in Amanat Al Asimah, followed by Taiz (12 studies), Aden and Al Hudaydah (7 studies each), and Dhamar and Ibb (7 studies each) (6 studies per governorate), followed by Abyan and Lahij governorates (2 studies for each governorate). Al Bayda, Marib, Sana’a, and Socotra each had one study. The governorates (Mahra, Saada, Amran, Raymah, and Shabwa) were deprived of all joint or independent studies, and seven studies were almost comprehensive for all governorates as shown in Figure 2, Table 2. The following subsections describe the details of each category.

**Table 1.** Shows a summary of studies according to the topics during the war years from 2014 to 2020 (infectious diseases, non-infectious diseases, blood-related diseases, injuries and accidents, oral and dental problems, health system, family and society studies)

No	Region	Study Design	Description	Date	Ref
Infectious diseases					
1	Taiz- Al Mahwit	cross-sectional study	Demographic, socio-economic, housing conditions, and personal hygiene information were obtained. Fecal samples were collected from 498 children and screened for intestinal parasites.	2020	(Al-Mekhlafi, 2020)
2	Taiz	cross-sectional study	The children’s demographic and socio-economic information and their KAP towards schistosomiasis were collected; then, A total of 406 children were screened for urogenital and intestinal schistosomiasis.	2020	(Alharazi and Al-Mekhlafi, 2020)
3	Al- Hodeidah	cross-sectional study	A demographic data of 400 children were obtained, and physical, laboratory measurements, and the necessary examinations were performed.	2020	(Alwajeeh, Abdul-Ghani *et al*., 2020)
4	Sanaa City	A cross-sectional study	A total of 813 aged 5 to 15 years asymptomatic school children whose antistreptolysin O test was negative were included. Throat swabs and blood samples were taken from each student.	2019	(Othman, Assayaghi *et al*., 2019)
5	Hajjah	Descriptive Study	A determine the prevalence of intestinal parasitoses and schistosome infections among 780 children, along with an assessment of praziquantel (PZQ) in the treatment of microscopy-confirmed cases of Schistosoma mansoni and Schistosoma hematobium.	2019	(Alharbi, Alwajeeh *et al*. 2019)
6	Al- Mahwit	Descriptive Study	The prevalence and distribution of parasitic intestinal infections among 200 primary school children (aged 7-15 years) were determined. Stool samples were collected and examined.	2016	(Alwabr and Al-Moayed, 2016)
7	Taiz- Ibb- Dhamar, Sana’a- Hudaydah	A cross-sectional study	The collected urine and fecal samples of 400 children from 5 different governorates were examined; the schistosomes were found in the samples. To test the S. mansoni and S. hematobium mitochondrial gene diversity in each sample, a fragment of the mitochondrial genome was extracted.	2015	(Sady, Al-Mekhlafi *et al*., 2015)
8	Sanaa City-Aden	Retrospective study	Aims: to determine the rates of influenza and non-influenza among patients with acute respiratory infection (SARI) by analyzing the data of 1811 patients from 2011-2016, where the rates of positive influenza viruses (type AB) and other viruses were calculated. The number of those admitted to intensive care and the number of deaths were also counted.	2019	(Al Amad, Al Mahaqri *et al*., 2019)
9	Al Hodydah- Al Mahwit	cross-sectional study	A total of 508 residents were sampled by a multi-stage random approach, data were collected, and blood specimens were screened for anti-Ov16 IgG4 using the SD BIOLINE Onchocerciasis IgG4 rapid tests.	2018	(Mahdy, Abdul-Ghani *et al*., 2018)
10	Sanaa	Cross-sectional study	An intestinal parasites and other related factors among 1218 children in rural regions were detected. Socio-demographic data and some behavioral factors, as well as stool samples, were taken to examine for parasites.	2016	(Al-Mekhlafi, Abdul-Ghani *et al*., 2016)
11	Al Bayda- Ibb- Dhamar	A clinic-epidemiological study	Aims: identifying various features and signs of leishmaniasis, as well as clinical manifestations for 152 confirmed cases from April to August 2013 at the Leishmaniasis Disease Diagnostic and Treatment Center.	2016	(Al-Kamel, 2016)
12	Taiz- Aden	A retrospective observational study	Aims: Evaluate the effect of RV vaccination on the epidemic diseases of acute gastroenteritis (AGE), and the characteristics of circulating RV strains, the prevalence of major pathogenic intestinal RV strains by antibiotic use was obtained on 5,691 children under 5 who had a history of diarrhea from 5 institutions between (2007-2011) and (2013-2014).	2015	(Banajeh and Abu-Asba, 2015)
13	Sanaa city	A record-based study	From 2011 to 2013, a 43 200 symptomatic patients were evaluated for helicobacter pylori (H. pylori) using H. antigens, antibodies, and parasites. A total of 1008 children (boy:675) (67 %), girls: 333 (33 %) were infected.	2014	(Bin Mohanna, Al-Zubairi *et al*., 2014)
14	Al- Hudaydah	Descriptive study	The epidemiological characterization and the epidemiological features of the Chikungunya outbreak had been highlighted.	2014	(Malik, Mnzava *et al*., 2014)
15	Taiz	An analytical descriptive study	Aims: Investigate the rate of Rotavirus infection, its genetic makeup, and the factors that influence this disease in children. From November 2006 to February 2008, a total of 795 samples of fecal matter were collected from kids (less than five years old) who had diarrhea. Stool specimens obtained from patients were tested using the enzyme-linked immunosorbent assay (ELISA) for the presence of rotavirus.	2014	(Al-Badani, Al-Areqi *et al*. 2014)
Non-Infectious disease					
1	Dhale, Al Hodeihah, Al-Jawf, Hadramoot, Hajjah, Ibb, Lahij, Ma’rib, Taiz)	Survey study	By conducting a cluster survey in nine governorates using the Global Trachoma Mapping Project systems from 2013 to 2015 and the risk factors for developing the pulmonary disease in children (1-9) years were assessed.	2018	(Ali Thabit, Al-Khatib *et al*. 2018)
2	Sanaa	A cross-sectional study	Identify the bacterial etiological agents and antibiotic sensitivity patterns of 150 patients with otitis media (OM). Bacteriological tests were performed to collect samples of ear discharge and identify bacterial strains using biochemical tests. Patients and parents also were filling out the questionnaire.	2016	(Bin Mohanna and Bahannan 2016)
3	Hadhramout Cancer, The data is from all of Yemen	descriptive retrospective study	The patterns childhood cancers between 2002 and 2014 were determined. The study was based on Hadhramout Cancer Registry secondary data. All cancer-diagnosed children under 15 years of age were included. The international system for classifying children’s cancer was used to categorize types of cancer.	2016	(Jawass, Al-Ezzi *et al*. 2016)
4	Sanaa city	descriptive study	The aim: Characterizing the species Leishmania isolated from pediatric bone marrows by sequence analyzes of the transcribed internal ribosomes.	2016	(Mahdy, Al-Mekhlafi *et al*. 2016)
5	Sanaa city	cross-sectional study before and after treatment	The aim: Determine the clinical and biochemical characteristics of adults and children with visceral leishmaniasis (VL) and the differences between them.	2016	(Al-Ghazaly and Al-Dubai 2016)
6	Socotra island	cross-sectional study	The aim: Determine CSOM and related DHI prevalence in the Socotra Island among students between the ages of 6 and 16. From 20 April 2011 to 20 June 2011 using a questionnaire, an otoscopic ear check, an audiometric hearing test, and DHI fork tuning tests.	2015	(Muftah, Mackenzie *et al*. 2015)
7	Hadhramout	A case-control study	A risk factors for diarrhea in 200 children under 5 years of age were examined between February and April 2013 in Maternity and Child Hospitals special, outpatient clinics, and primary health care centers.	2015	(Bahartha and AlEzzi, 2015)
8	Sanaa city	a case report	A 13-year-old girl with an 8-yr history of severe rickets, which caused multiple bone defects were described.	2014	(Al-Sharafi, Al-Imad *et al*., 2014)
9	Sanaa city	A retrospective analysis study	The rates for the introduction of Hib vaccine among children aged 2–60 months for very serious pneumonia and all-over-meningitis and the results of bacterial meningitis surveillance from the main hospital for children in Sana’a between 2000 and 2010 were conducted.	2014	(Banajeh, Ashoor *et al*., 2014)
10	Sanaa city	A retrospective study	The prevalence of hearing impairment among primary school students was estimated. 2200 children aged 6 to 9 years attending 12 elementary schools were tested using a portable audiometer with an Otoscopy Tone.	2014	(Al’shardzhabi and Tsygankova, 2014)
Blood diseases					
1	Aden	A retrospective, descriptive study	The study was conducted on all children aged 16 years or younger. Who received blood transfusion therapy during their hospital admissions in the pediatric department at Al-Sadaqa Teaching Hospital, for a period of one year.	2020	(Al-Saqladi, Maddi *et al*., 2020)
2		Descriptive analytical study	The aim: Describe hematologic features of adults and children with Visceral Leishmaniasis, including peripheral blood and bone marrow aspiration(VL).	2017	(Al-Ghazaly, Al-Dubai *et al*., 2017)
3	Region not specified	An experimental, analytical study	The aim: investigate the possible association of polyps in these genes with alleles in a sample of children. 289 children were genotyped with seven single-nucleotide polymorphisms (136 cases and 153 controls).	2017	(Al-Absi, Razif *et al*., 2017)
4	Sanaa	Case-control study	The aim: Examine the association of ARID5B variants in children with acute lymphoblastic leukemia. In 289 children, of whom 136 have acute and 153 controlled lymphoblastic leukemia, a total of 14 ARID5B single-nucleotide polymers (SNP) were detected.	2017	(Al-Absi, Noor *et al*., 2017)
5	Al- Hodeidah	A cross-sectional study	In the study of the hematological characteristics findings, adults and children with Visceral Leishmaniasis all patients with visceral Leishmaniasis had anemia, (87%) leukopenia, (89%) neutropenia, (94%) thrombocytopenia, (89%) eosinopenia, (72%) pancytopenia and (28%) had bicytopenia. In bone marrow examination, 40 (85%) showed hypercellularity, (94%) eosinopenia, (51%) dyserythropoietic, (47%) lymphocytosis, (17%) plasmacytosis, (57%) decreased iron stores and (43%) showed decreased sideroblasts	2016	(Abdul-Ghani, Mahdy *et al*., 2016)
6	Aden	This cross-sectional study	The aim: Describe patterns and correlation with age, sex, and other risks of hepatobiliary complications of SCD patients. 106 SCD patients admitted from January to June 2009 were assessed. All patients have a thorough history, thorough investigation, essential laboratory research, and abdominal ultrasound (including full blood counts, liver and viral marker tests).	2014	(Qhalib and Zain, 2014)
7	Aden	A descriptive, diagnostic study	The aim: Estimate the prevalence of beta-hemolytic (GAS) group A and non-GAS infections among children suffering from acute pharyngotonsillitis using fast GAS anti-SSD (RADT) test and/or GAS culture from a throat swab in school-age children.	2014	(Ba-Saddik, Munibari *et al*., 2014)
Oral and dental problems					
1	Sanaa	Descriptive study	Prevalence of dental caries was evaluated in children in cities and rural, and correlations between caries and fluoride levels were examined in drinking water, aging, gender, and residence. 17 599 children aged (6–12 years) were studied (9623 boys and 7,976 girls) using decay-filled teeth and decay-filled teeth indexes to evaluate dental caries.	2018	(Al-Akwa and Al-Maweri, 2018)
2	Sanaa	Descriptive study	Prevalence and severity of dental erosion in 668 groups of children and adolescents are determined and a proposed modified-simplified version of an erosion partial recording system (EPRS) is compared clinically (EPRS-M).	2017	(Al-Ashtal, Johansson *et al*., 2017)
3	Dhamar	A cross-sectional study	The aim: Evaluating the prevalence of premature primary tooth loss in the age group 5–10 years. There were 185 children, 91 boys, and 94 girls. An experienced examiner under adequate artificial light conducted the dental examination.	2016	(Murshid, Al-Labani *et al*., 2016)
4	Dhamar	A Cross-sectional Study	The aim: Measuring gingivitis prevalence and severity among students aged 6 to 12. A total of 663 children were examined (310 kids aged 6 years and 353 kids aged 12 years). Gingival health has been evaluated on six Ramfjord teeth by means of the plaque index (PI), the computational index (CI), and the gingival index (GI).	2016	(Amran, Alhajj *et al*., 2016)
5	Sanaa city	Descriptive study	Researchers studied the prevalence of various oral disorders in children aged between the ages of 4 and 12 years using the examiner used disposable tongue blades.	2016	(Basalamah and Baroudi, 2016)
6	Sanaa city	Descriptive survey study	Assessment of the oral health status and treatment needs of children with disabilities attending special schools in Sana’a.	2015	(Al-Maweri and Zimmer, 2015)
7	Sanaa city	Analytic scout study	Evaluation of the carriage rates, counts, and species distribution of Candida in the saliva of 180 children 6 to 12 years old. The simplified oral hygiene index and dmft/DMFT indices were used to assess oral hygiene and the use of caries. 4 Candida species were detected and quantified with CHROMagar Candida medium in non-stimulated saliva.	2015	(Al-Hebshi, Al-Maswary *et al*., 2015)
8	Sanaa city	A case-control study	To assess the prevalence of injuries to the mouth and jaws in children with autism. This research study incorporates 42 children of both male and female children, ranging in age from five to sixteen, and 84 healthy control groups of the same age and gender. standardized criteria were evaluated for oral lesions. Dental caries, gingival health, and oral hygiene status were evaluated using the Gingival Index (GI) and Plaque Index (PI), respectively.	2014	(Al-Maweri, Halboub *et al*., 2014)
9	Sanaa city	cross-sectional study	Assess the prevalence of dental caries and the treatment needs of 96 children with DS and to examine the association with different socio-demographic and clinical variables between these outcome results. Data was collected using a clinical observation questionnaire. Teeth and treatment needs have been recorded according to the recommendations of the WHO.	2014	(Al-Maweri and Al-Sufyani, 2014)
10	Sanaa city	A cross-sectional study	To determine the children’s oral hygiene and gingival health. 101 children with special needs were observed in special education school’ The calculus index (CI), gingival index (GI), and plaque index (PI) were used to assess oral hygiene and gingival health.	2014	(Al-Sufyani, Al-Maweri *et al*., 2014)
11	6 unspecified Yemeni governorates.	Descriptive survey study	Aim: to estimate the orthodontic treatment need in a sample of 3003 children aged 12-year-old using the dental esthetic index (DAI).	2014	(Al-Zubair, 2014)
12	Sanaa city	A comparative descriptive study	To evaluate the prevalence of oral lesions and dental caries in orphan children. The total of 404 child, with an equal number orphans and non-orphans were tested by an OML and decayed dental evaluation systems. The clinical test included an OML evaluation based on international standard diagnostic criteria and a decayed dental evaluation.	2014	(Al-Maweri, Al-Soneidar *et al*., 2014)
Accidents and injuries					
1	Sanaa city	A cross-sectional school-based study	A total of 1140 students (558 girls and 582 boys) participated in this study to determine unintentional injury among school children. Of all students, 550 (48.2%) students reported unintentional injuries during the last 12 months.	2018	(Alshahethi, Al Serouri *et al*., 2018)
2	Sanaa city	A prospective observational case series study	To elucidate causes of ocular trauma in children younger than 17 years of age during the Eid festivities in Sana’a in 2016. All children up to 17 years of age presenting with ocular trauma were included in the study.	2020	(Aldoais, Bamashmus *et al*., 2020)
Health System					
1	A comprehensive study of regions	Surveying and building a new model for health facilities	The researchers extracted geospatial coordinates and data availability of services for health facilities from an assessment of a monitoring system for health resources and services conducted by the World Health Organization and the Yemeni Ministry of Health and Population.	2020	(Al-Mudhwahi, 2015)
2	Taiz- Lahij- Aden	Survey study	Aim: to explain some of the reasons for the weakness of the health system, as it showed that community health workers provide comprehensive camp management services in difficult and insecure conditions due to the ongoing conflict.	2020	(Miller, Zunong *et al*., 2020)
3	Hajjah	retrospective study	A total of 976 newborns; 506 preterm newborns (51.8%) and 470 term newborns (48.2%) were evaluated. Over half, 549 (56.3%) newborns were admitted within 24 h after birth, and 681 (69.8%) newborns traveled for over 60 min to arrive at the NICU.	2020	(Eze, Al-Maktari *et al*., 2020)
4	Aden – Sanaa city – Taiz	A case study	To examine how reproductive, maternal, newborn, child, and adolescent health and nutrition (RMNCAH + N) services have been delivered since 2015 and identified factors influencing the implementation of these services in three governorates.	2020	(Tappis, Elaraby *et al*., 2020)
5	A comprehensive study of regions	Descriptive Study	Aim: to provide a description of the impact of the 2015 war on the immunization of children under five years of age by analyzing data on vaccination coverage from 2012 to 2015 from the expanded national program.	2019	(Torbosh, Al Amad *et al*., 2019)
6	Aden	An analytical study	The researchers conducted the study on 1,000 children who lived an entire pregnancy period of 37–42 weeks, including 488 males and 512 females within or 24 h of birth from 2002–2003.	2020	(Ba-Saddik and Al-Asbahi, 2020)
7	Yemeni countryside	A reference study based on a previous representative survey in 2008-2009.	Using the principal component analysis (PCA) proposed three indicators (wealth – education – housing quality) to classify rural Yemeni women, and whether it is possible to predict through these indicators using services derived from taking care of mother and child health in the Yemeni countryside.	2019	(Alosaimi, Nwaru *et al*., 2019)
8	Sanaa city – Ebb	Descriptive study	The researchers obtained data from the Yemeni Nutrition Monitoring Program, which targeted more than 4,000 families and contained more than 5,000 children under five years of age.	2019	(Dureab, Al-Falahi *et al*., 2019)
9	A comprehensive study of regions	An analytical study	Analyzing the outbreak of diphtheria during the first three years of the war when the Yemen health system failed to cover immunization. It relied on analyzing data from publications taken from reports of diphtheria. The impact of the conflict was studied each year on the coverage of vaccination against diphtheria.	2019	(Dureab, Al-Sakkaf *et al*., 2019)
10	Abyan	A community-based cross-sectional study	Aim:to assess the prevalence of undernutrition among 1292 children under the age of 5 years and then use the results of the CIAF assessment using traditional indicators and the composite index of anthropometric.	2019	(Al-Sadeeq, Bukair *et al*., 2019)
11	Dhamar	qualitative study qualitative in-depth interviews	Aim: to test the practices that the program planned to test and evaluate (MIYCN) and (FP) to present proposals for new practices that included visiting 32 mothers and 16 fathers of children less than two years of age.	2019	(Mohamed Assabri, Cooper *et al*., 2019)
12	A comprehensive study of regions	Descriptive study	Researchers used various data sources to provide estimates of 12 maternal and child health indicators in 2016 on child immunization, mother and child nutritional status, and the change in these estimates for the period 2013–2016 based on new variables including change in GDP and the burden of war.	2018	(El Bcheraoui, Jumaan *et al*., 2018)
13	Taiz	Descriptive survey research	Aim: to determine the coverage rate of HBV vaccine and assess the vaccine protective response among 227 children under five years old in rural areas using the questionnaire Serum samples were tested for anti-HBs antibodies.	2017	(Alssamei, Al-Sonboli *et al*., 2017)
14	Sanaa	A prospective study	Measuring the rate of therapeutic deficiencies in the 11 primary care centers among SAM children. For data collection on admission and after 8 weeks of OTP acceptance, a pre-tested questionnaire was used.	2017	(Al Amad, Al-Eryani *et al*., 2017)
15	A comprehensive national survey	Study using secondary data	An assessment of the role of integrated outreach activities in improving the nutritional condition of under-five children in terms of efficiency of outreach services in order to improve their access to basic and social services and their economic opportunities.	2015	(Al-Mudhwahi, 2015)
16	Abyan	A cross-sectional study	The study examined urine samples and interviewed 696 children (mean age was 12.5 years) to compare urinary egg presence as a standard diagnosis, a questionnaire on morbidity, and a streak of urine reagents were assessed as a residential testing tool for school children.	2014	(Bassiouny, Hasab *et al*., 2014)
	**Family and social studies**				
1	Unspecified random sample	Descriptive study	A mother’s religiosity as a major cultural factor in the impact of harsh physical parenting on the behavioral problems of children was evaluated. Data were collected from various sources, including discipline monitoring, short articles based on religiosity, and a questionnaire was conducted.	2019	(Alsarhi, Prevoo *et al*., 2019)
2	Dhale- Taiz- Ibb- Al- Hodeidah	Study using secondary data from a partial cross-sectional scan.	The researchers analyzed data of 7341 women aged of (15–49) years carried out by UNICEF in the years 2008-2009.	2019	(Alosaimi, Essén *et al*., 2019)
4	Dhale- Taiz- Ibb- Al- Hodeidah	Study using secondary data from a partial cross-sectional scan	By using factor analyses to build the SES indicators for 7,295 women of reproductive age. Data from a sub-national household survey in four governorate were collected in 2 years 2008–2009. Logistical regression models have been adapted to estimate the associations among SES indexes with spontaneous abortion, maternal death, mortality neonatal, and infant mortality.	2016	(Alosaimi, Luoto *et al*., 2016)
5	A comprehensive study of regions	Descriptive study	The relationship between undernutrition in rural children and the adequacy of adult caregivers was examined. Mother and Child Health according to data of 2013 for 3,549 children under the age of 5 years were investigated. The food status was assessed in accordance with the WHO child growth standards by the presence of underweight, stun and waste.	2016	(Al-Sobaihi, Nakamura *et al*., 2016)
6	Taiz- Ibb, Dhamar,- Sana’a - Hudaydah	A cross-sectional study	Urogenital and intestinal schistosomiasis has been examined in 400 children in ten districts of rural areas. Then an interview with parents, information on demographic, socio-economic information, and their KAP regarding schistosomiasis was collected using a questionnaire.	2015	(Sady, Al-Mekhlafi *et al*., 2015)
7	Sanaa city	focus group study	The data of 31 moms with at least one child younger than 5 years with a history of fever, diarrhea, cough, or difficulty breathing between 1 and 6 April 2014 were obtained and analyzed using micro-interlocutor analysis.	2014	(Webair and Bin Ghouth, 2014)

Table (2) shows the number of private and joint studies in each governorate and compares it to the population.

**Table 2.** Showing the number of independent and joint studies in each governorate and comparing it to the population

			Type of study	
Name	Population (2013)	Number of studies	independent	joint
Abyan	658 824	2	0	2
Aden	1 087 653	7	5	2
Al Bayda	835 683	1	0	1
Al Hudaydah	3 774 914	7	3	4
Al-Jawf	663 147	1	0	1
Al Mahrah	400 000	0	0	0
Al Mahwit	732 360	2	0	2
Amanat Al Asimah (Sanaa City)	1 174 767	21	18	3
Amran	1 123 651	0	0	0
Dhale	602 613	3	0	3
Dhamar	1 697 067	6	3	3
Hadramaut	1 329 085	1	0	1
Hajjah	1 887 213	3	2	1
Ibb	3 911 070	6	0	6
Lahij	926 291	2	0	2
Ma’rib	504 696	1	0	1
Raymah	502 505	0	0	0
Sa’dah	987 663	0	0	0
Sana’a	2 279 665	1	1	0
Shabwah	651 509	0	0	0
Socotra	60 000	1	1	0
Taiz	4 554 443	12	3	9
	30,344,819			

Ministry of Public, Population *et al*. (2015).

### Infectious diseases

The spread of epidemics was associated with the continuation of the war in Yemen. For example, Yemen was exposed to two waves of cholera during the war, and it was the largest disease outbreak in modern history (Blackburn, *et al*., 2020). Previous research indicates that the prevalence of infectious diseases such as *Hymenolepis nana*, schistosomiasis, and malaria is high among Yemeni children who have little knowledge about them. (Al-Mekhlafi, 2020; Alharazi and Al-Mekhlafi, 2020; Alwajeeh, Abdul-Ghani *et al*., 2020). In a survey on Pharyngeal Streptococcus pyogenes carriage rates in healthy school children, females were shown to be more susceptible (Othman, Assayaghi *et al*., 2019). In studies of intestinal parasites, 75%–85% of primary school children were infected with intestinal parasites such as *Entamoeba histolytica* (27.8%) and *Hymenolepis nana* (12.2%) (Alwabr and Al-Moayed, 2016, Alharbi, Alwajeeh *et al*., 2019). In 5 Yemeni governorates, 400 children were surveyed to search for species diversity of Schistosoma, and researchers found that 31.8% of those were excreting schistosomal eggs in the urine or feces(Sady, Al-Mekhlafi *et al*., 2015). Between 2011 and 2016, 1811 SARI patients were admitted, with 78% being under the age of 15 and a high percentage having non-influenza viruses (Al Amad, Al Mahaqri *et al*., 2019).

In a test for *Onchocerca volvulus* infection in the Tihama area, it was found that there is an infectious spread in different proportions (Mahdy, Abdul-Ghani *et al*., 2018). In Sana’a, parasites were studied, and the researchers discovered a relationship between infectivity and a variety of parameters like hygiene, awareness, and parental education level (Al-Mekhlafi, Abdul-Ghani *et al*., 2016). In a clinical, epidemiological leishmaniasis study in central Yemen, 94.1% were rural and the Bayda area is the most endemic region (Al-Kamel, 2016).

A study analyzed surveillance data on 5,691 children who visited the hospital and discovered that vomiting and diarrhea symptoms varied significantly between children. Prior to rotavirus vaccination, rotavirus acute gastroenteritis prevalence peaked in October, November, and December at 58.8%, 69.5%, and 56.4%, respectively (Banajeh and Abu-Asba, 2015). Similarly in Taiz, 45.2% were Rotavirus-positive, and the most prevalent genetic constitution was G2P (55%) (Al-Badani, Al-Areqi *et al*., 2014). Between 2013 and 2014, the variance became less defined as a result of the introduction of the rotavirus vaccination, which resulted in a few cases of rotavirus acute gastroenteritis (Banajeh and Abu-Asba, 2015). According to the study on *H. pylori* and parasite-infected children, the estimated prevalence of symptomatic children among 43 200 children varies from 3 to 15 years (Moqbel). While *H. pylori* infections were prevalent in 83% of 6–8 year olds and 52% of 12 to 15 year olds, giardiasis was prevalent in 10% and amoebiasis was prevalent in 25% of children (Bin Mohanna, Al-Zubairi *et al*., 2014).

A total of 10 715 suspected cases of chikungunya were reported from the Al Hudaydah governorate between 2010 and 2011, indicating the country’s first outbreak. An overall attack rate ranged between 22 and 24 per 1000 cases (Malik, Mnzava *et al*., 2014). The prevalence of infectious diseases among Yemeni children constitutes one of the important conflict problems that must be addressed promptly.

### Non-infectious disease

A survey of risk factors for lung disease was conducted in several governorates, and the researchers concluded that the prevalence of trachomatous inflammation-follicular disease was 10% in children aged 1–9 years in two evaluation units and that there was an effect by gender, the number of children in a family, and the family’s habit of defecating in the open were all independently associated with an increased risk of developing burnout (Ali Thabit, Al-Khatib *et al*., 2018). Additionally, some risk factors are linked to diarrhea and other children’s diseases like crowded housing and incomplete child vaccination for children under five years old (Bahartha and AlEzzi, 2015).

According to one study, 150 children aged 1–15 years were diagnosed with otitis media (85 males and 65 females) (Bin Mohanna and Bahannan, 2016). To screen for chronic suppurative otitis media (CSOM), a total of 686 children were diagnosed with both CSOM and associated hearing problems, indicating that there was a significant correlation between demographics and CSOM status. Logistic regression identified four important independent contributing factors: history of ear discharge in the last 12 months; swimming in local pools; recurrent respiratory tract infection more than three times per year (Muftah, Mackenzie *et al*., 2015). In an attempt to estimate the prevalence of impaired hearing loss, as many as 11.6% of the total number of children failed to pass the test and the percentage decreased to 10.6% after cleaning the external auditory canal. The prevalence of hearing impairment among Yemeni elementary school children was shown to exceed compared to the children in developed countries (Al’shardzhabi and Tsygankova, 2014).

To understand the species which cause visceral leishmaniasis, smears were collected and found positive for leishmania species amastigotes. The percentage of children among them was 40.4%, with a mean age of 7.1 years ± 4.7. Fever, pallor, splenomegaly, and hepatomegaly were the most common clinical findings, and hypoalbuminemia and hyperglobulinemia were common biochemical abnormalities (Al-Ghazaly and Al-Dubai, 2016). In a study to describe the hematological characteristics findings, adults and children with visceral leishmaniasis had anemia, and most of the blood components were deficient from the normal count (Al-Ghazaly, Al-Dubai *et al*., 2017). One study concluded that very severe pneumonia was still the leading cause of severe morbidity and death for young children, particularly those aged < 12 months (Banajeh, Ashoor *et al*., 2014). To identify the prevalence of severe rickets, the patients were fed on a gluten-free diet, vitamin D supplements, calcium, and iron replacement. Laboratory investigations showed some improvements from 5 months earlier which reflects the malnutritional status of Yemeni children who can be saved with simple intervention (Al-Sharafi, Al-Imad *et al*., 2014).

### Blood diseases

A study conducted in one of the hospitals in Aden city, out of 217 children with SCD (define SCD), the commonest indications for transfusion were anemic crises (41.1%), vaso-occlusive crises (VOC) (13.8%), VOC with an anemic event (11.3%), acute chest syndrome (8.7%), and stroke (7.3%) (Al-Saqladi, Maddi *et al*., 2020). Hepatobiliary complications increased significantly with age; amongst those frequently admitted and/or suffering from a blood transfusion were significantly higher (Qhalib and Zain, 2014). A study found a deficiency of enzyme activity of glucose 6-phosphate dehydrogenase (G6PD) toward 60% of normal activity among 12% of children residing in Hodeidah Governorate. In malaria-endemic areas, 2%–3% having severe G6PD deficiencies is not uncommon. More extensive genotyping is required to comprehend the local infectious diseases caused by circulating strains (Ba-Saddik, Munibari *et al*., 2014). A study of tonsillopharyngitis among children found that more extensive genotyping is required to comprehend the local infectious diseases caused by circulating strains of beta-hemolytic (GAS) (Ba-Saddik, Munibari *et al*., 2014).

A total of 406 childhood cancers were detected in a study of malignancies that accounted for 8.5 % of all cancers reported between 2002 and 2014. The mean age was found to be 7.34 ± 4.18 years with 59.1% male children. The most common group of malignancies was hematological malignancies accounting for 47% followed by nervous system malignancies (15%) (Jawass, Al-Ezzi *et al*., 2016). Under additive genetic models, the researchers discovered that nine single nucleotide polymorphisms (SNPs) were associated with acute lymphoblastic leukemia. (Al-Absi, Noor *et al*., 2017). A study conducted on the possible association between polymorphisms in genes and alleles reported that the IKZF1 SNP and the CDKN2A polymorphism were significantly associated with acute lymphoblastic leukemia in children (Al-Absi, Razif *et al*., 2017).

### Oral and dental problems

Several studies related to oral and dental problems were all mostly conducted in the capital city of Sana’a in the Dhamar governorate. One survey found a very high prevalence of dental caries among school children 67.6%. Children residing in urban districts had significantly higher mean scores of DMFT/deft which is a general indicator of dental health status than those in rural areas. A significant negative correlation between caries experience and fluoride level was also found (Al-Akwa and Al-Maweri, 2018). A study on 6163 kids in the age bracket of 5–19 year olds discovered that the prevalence of dental erosion in children aged 5–6 was 6.8%, 3% in 13–14 year olds, and 14.6% in those aged between 18 to 19, with a higher prevalence in girls. Dental erosion was common among children and older teenagers and highest among older girls but less common among younger teenagers. The tested accuracy of EPRS-M is a gold standard in the quick detection tool in future dental erosion research (Al-Ashtal, Johansson *et al*., 2017). Researchers found 40.54% premature loss of primary teeth among 185 children with the second lower left molar as the most commonly missing dental tooth. To promote oral health, children need to implement educational and preventive programs (Murshid, Al-Labani *et al*., 2016). A total of 663 children were examined from 10 public primary schools in Dhamar City, and it was found that poor oral hygiene and mild gingivitis were highly prevalent among Yemeni school children. An early assessment and intervention of gingivitis and periodontitis could minimize the chance of tooth loss (Amran, Alhajj *et al*., 2016). The overall prevalence of oral anomalies among Yemeni children was found to be 15.1% at a male-to-female ratio of 3.2:1, with 7 to 12 year olds having the highest prevalence. Tooth hypoplasia and hypo calcification were the most common dental anomalies related to hard tissues, while fissured tongue was the most common soft tissue anomaly (Basalamah and Baroudi, 2016).

One study assessed the oral health status and treatment needs of children with disabilities attending special schools in Sana’a and the mean dmft, and DMFT scores of the total population were 4.27 and 1.90, respectively, indicating that children with disabilities have a high prevalence of dental caries and poor oral hygiene (Al-Maweri and Zimmer, 2015). Another study on the assessment of oral health among autistic children in Sana’a city and 42 children with autism revealed a higher proportion of fistulae (9.5% vs. 2.4%), ulcerative lesions (7.1% vs. 1.2%), gingival hyperplasia (4.8% vs. 0.0%), and cheilitis (4.8% vs. 2.4%), and the mean dmft score was higher in children with autism than in controls (5.23 vs. 4.06; *P* < 0.001). While children with autism revealed poorer oral hygiene than controls, the majority had gingivitis (Al-Maweri, Halboub *et al*., 2014). One research showed that 93.8% of the subjects had dental caries; overall DMFS, DMFT was 10.35, 4.44, 4.32, and 2.45, respectively. Dental caries was also reported in children with Down syndrome aged between 6 and 15 years (Al-Maweri and Al-Sufyani, 2014).

To find salivary Candida species carriage patterns and their relation to caries experience among children, Candida was detected in 60% of the children and *C. albicans* accounted for 60% of the isolates. A novel finding was that a significant proportion (38%) of the carriers harbored two or more species, which for the first time allowed the identification of four age-dependent carriage patterns (clusters). Another somewhat new observation was that carriage at ≥ 1000 CFU/ml in particular significantly correlated with caries in primary and permanent dentitions (r = 0.23 and 0.18, respectively) as well as a caries-active status (OR = 6.9) (Al-Hebshi, Al-Maswary *et al*., 2015). Researchers discovered that 36.6% school children aged 12 years require orthodontic treatment, and nearly 1 out of 5 school children had a dental esthetic index of 31 points or above, implying that that the need for orthodontic therapy is highly desirable or mandatory (Al-Zubair, 2014).

A study was conducted on the assessment of the prevalence of oral mucosal lesions among the institutionalized orphan children in Sana’a city, with samples of 202 orphan male children matched with 202 non-orphaned students, and the majority of them were 12–15 years old. Nine types of lesions in orphans were reported, the most frequent lesions being tongue fissure (24.3%), labial herpes (7.9%), and traumatic sores (2.5 percent). Herpes labialis occurred in orphans substantially higher than in controls (*P* < 0.01). The prevalence of dental cariousness among orphans was significantly lower (84.7%) than that of non-orphans (89.61%; *P* = 0.136). In orphans, the mean dmft value of the controls (2.28 vs. 3.82; *P* = 0.001) was significantly lower.(Al-Maweri, Al-Soneidar *et al*., 2014). All of these findings strongly suggest that orphans and children with low education levels and low income have a bad effect on children’s oral health and that efforts must be made to resolve the conflict problem, which increases the percentage of orphans and low social life status.

### Accidents and injuries

This section included a minimal number of researches despite the great injuries caused by the war among children that negatively affect knowledge of current status.

One of the studies in 2018 researched about rate and pattern of unintentional injuries among 9–12 grade school children and their associated factors. Among the sample population, 550 students (48.2%) reported unintended injuries within the past 12 months from the date of examination. More injuries were reported in males compared to females (odds ratio = 1.6). The study found an association between a child’s loss of one or both parents with an increased risk of injury (odds ratio = 1.7). There was also an association between parents’ old age or death and an increased risk. The proportion of girls who were infected at home was more than boys (58.9%: 30.9%). Most than two-thirds of the injuries affected the upper or lower extremities (64.9%) and the majority of students (98.4%) recovered from injury, while 1.6% of injuries resulted in permanent disability (Alshahethi, Al Serouri *et al*., 2018). Medical research was conducted in two hospitals in Sana’a in order to uncover the potential causes of ocular injury. All the subjects were males between 4 and 15 years old, and the injuries among the children involved the right eye (52.5%) and the left eye (47.5%). The majority of injuries (n = 152, 95.0%) had occurred in the street, while eight (5.0%) had happened at home, and the most frequent cause of injury was reported to be fireworks mishaps (Aldoais, Bamashmus *et al*., 2020). The preceding findings indicate that parent loss significantly increased during times of conflict and is substantially connected with child injury. Additionally, the possession of firearms and war objects by individuals has a detrimental effect on the occurrence of injuries among children.

### Health system

A study on the efficiency of the role of integrated outreach activities between 2004 and 2014 years was published in 2015 that included two components of the health system viz. health intervention coverage levels and protection of risks at the financial level. The millennium development goals including immunization coverage, services management of infancy, reproductive health, and control of dangerous diseases were Yemen’s intervention coverage indicators at that time and had progressed very well. However, children aged under five were still highly malnourished in the country at that time (Al-Mudhwahi, 2015).

Yemen’s conflict has had a significant impact on the health system, with only 51% of health institutions fully operational and 19.7 million people lacking access to health care (Miller, Zunong *et al*., 2020). All working groups agree that Yemen’s health system has collapsed. In 2018, researchers discovered that around 8.8 million people needed more than 30 min to access a primary health facility and more than an hour for 12 million individuals to get to the hospital (Eze, Al-Maktari *et al*., 2020). Furthermore, the safety of health professionals has become an impediment to providing services to the population in war zones, with travel challenges identified as the greatest threat to health workers’ safety (Miller, Zunong *et al*., 2020).

A previous study was performed to describe the neonatal intensive care unit (NICU) outcomes in a complex humanitarian conflict setting in Hajjah governorate throughout the years 2017–2018 and they found that; the most frequently diagnosed conditions were prematurity problems (34.9%), perinatal asphyxia (34.4%), neonatal jaundice (18.8%), and neonatal sepsis (16.1%). Eighty-three percent of preterm newborn deaths happened in newborns who traveled more than an hour to reach the NICU (Eze, Al-Maktari *et al*., 2020). At the time of crisis and epidemics, humanitarian response efforts were redirected toward other services, deprioritizing the neonatal health system (Tappis, Elaraby *et al*., 2020).

Another study enrolled 339 children who were very malnourished. Among them, 42% were successfully treated, 55% were dismissed as defaulters, and 3% were moved to other treatment facilities. A high default rate was substantially associated with factors such as inadequate services, employee and system dissatisfaction, ambulatory therapy program treatment, and acceptance of staff services. The study advocated expanding outpatient therapeutic program OTP services and providing OTP employees with training in severe acute malnutrition treatment methods (Al Amad, Al-Eryani *et al*., 2017). About the coverage of vaccines, one study found an increase from 2012 to 2014 in the national coverage for the Penta-3 vaccine (82% in 2012 vs. 88% in 2014) and measles vaccine (70% in 2012 vs. 75% in 2014). The coverage was still below the national target (≥95%). The year 2015 witnessed a marked decline in the national coverage compared with 2014 for the measles vaccine (66% in 2015 vs. 75% in 2014) (Torbosh, Al Amad *et al*., 2019). Vaccine coverage decreased between 2013 and 2016 among children aged 12–23 months, and the largest decrease was by 36.4% for the first dose of measles vaccine in Aden among children under the age of five. The rate of diarrhea incidence was 7.0 attacks per person per year (El Bcheraoui, Jumaan *et al*., 2018). The coverage rate of the hepatitis B virus HBs vaccine among children was 87.3%. A total of 72.2% children responded to the vaccine with an anti-HBs level ≥ 10 IU/L, while 27.8% of the children had nonprotective anti-HBs levels of <10 IU/L (*P* = 0.003). The rate of coverage of HBV vaccine in rural areas was excellent, while the protective rate against HBV infection was moderate.(Alssamei, Al-Sonboli *et al*., 2017).

Researchers used the principal component analysis to assess the socio-economic indicators and proposed wealth, education, and housing quality as the key maternal health indicators. Social and economic disparities should be taken into account in planning maternal and child health interventions (Alosaimi, Nwaru *et al*., 2019). According to data from the Yemeni Nutrition Monitoring Program from Sanaa city and Ibb governorate, the researchers found that 13.3% of the children suffer from global acute malnutrition, 8.4% suffer from moderate acute malnutrition, and 4.9% suffer from severe acute malnutrition. The researchers also found a relationship between malnutrition and other diseases such as measles, diarrhea, and fever, and that most families relied on buying food from the market (Dureab, Al-Falahi *et al*., 2019). Three years after the onset of war, an analysis on the outbreak of diphtheria was conducted, which demonstrated the inability of the health system to cover immunization was a major reason for the outbreak. In the districts that were experiencing ongoing conflict, the risk of an outbreak increased by 11-fold (Dureab, Al-Sakkaf *et al*., 2019).

An assessment of undernutrition was perfomed using the composite index of anthropometric failure (CIAF) among children aged < 5 years in rural Yemen. CIAF identified undernutrition in 70.1% of children, while conventional anthropometric indices revealed 38.5% stunting, 39.9% wasting, and 55.1% as underweight. According to CIAF, 21% had a single anthropometric failure, and 49.2% exhibited multiple failures. Stunting index, wasting index, and underweight index were 0.55, 0.57, and 0.79, respectively (Al-Sadeeq, Bukair *et al*., 2019).

To evaluate the extent of the population’s response to health education and changing negative habits in some countryside, it was found that wives and husbands were able to try to adopt new practices after one counseling visit. Most of the practices were successfully adopted by the respondents, and some mothers were also successful in adopting proper breastfeeding practices. Parents need more counseling and reinforcement to continue to adopt new health practices (Mohamed Assabri, Cooper *et al*., 2019). Urinary schistosomiasis has been confirmed by a quick diagnosis of schistosomiasis in 126 out of 696 children using a simple questionnaire and urinary examination using microhaematuria, which is used to identify individuals and communities infected with *Schistosome hematobium* (Bassiouny, Hasab *et al*., 2014).

### Family and social studies

The family and society greatly affect the development of children and the formation of their personalities, and this influence remains with the individual throughout his life. The most important health care services that internally displaced people IDPs need are women’s health services, because women, the elderly, and children are the most affected by displacement worldwide. The physiological role of women, poverty and deprivation, lack of health insurance, or insufficient information hinder women from adequately benefiting from health care services. Therefore, displaced/refugee women often face more problems related to women and reproductive health than locally residing women. Among the most important of these problems is the lack of family planning services, unwanted pregnancies, abortion, and obstetric complications (Döner and Şahin, 2021).

One study that examined mothers’ religiosity as a major cultural factor in the impact of harsh physical parenting on the behavioral problems of children showed that there was no direct correlation between harsh physical parenting and maternal religiosity and children’s behavioral issues. However, it was found that the positive association between harsh physical parenting and children’s behavior problems is stronger when parents are more religious (Alsarhi, Prevoo *et al*., 2019). A study done on female genital cutting (FGC) behavior in four governorates discovered that FGC was prevalent in 48% mothers, while daughters’ FGC was 34%. Almost 45.8% of the women surveyed believe that the FGC practice should be discontinued. Higher odds of FGC practice and positive attitude towards it were associated with older age, family marriage, and lower tertiles of wealth and education indices. Early marriage was also associated with increased odds of FGC practice (*P* < 0.01)(Alosaimi, Essén *et al*., 2019). Three socio-economic status (SES) (wealth, education, and quality of housing) indices were extracted in four provinces; capturing household attributes for 7295 in reproductive age were extracted; higher tertiles for all indices were inverse of spontaneous abortion. Wealth and education indices were inversely linked to infant mortality, neonatal, and mortality. (Alosaimi, Luoto *et al*., 2016).

Adult caregiving has been related to childhood malnutrition, according to a study. Baseline survey of mother and child health conducted a study in 2013 to understand the situation of adult caregiving. Researchers recorded and analyzed the data on children’s height and weight from a sample of 3,549 children under the age of five who lived in rural areas. Three measurements (wasting, stunting, and low weight-for-for-age) were applied to determine a child’s nutritional status in accordance with the standards of the WHO. They found 52.2% of children are wasted, 66.7% are underweight, and 11.3% are undernourished. Keeping children out of labor while receiving treatment for stunting and underweight, as well as giving them care, related to the lower risk of these children being stunted and under weight (Al-Sobaihi, Nakamura *et al*., 2016).

To find out how the family deals with schistosomiasis, studies were carried out among 250 households from ten rural districts. A total of 31.8% of children excreted schistosome eggs in urine or feces (8.0% *S. mansoni* and 22.5% *S. hematobium*). 92.4% of respondents had known schistosomiasis and knew about the transmission, signs, symptoms, and prevention. Multiple logistic regression analyses revealed that the level of the history of schistosomiasis and education were the most important factors associated with the knowledge, attitude, and practices concerning schistosomiasis among this population (Sady, Al-Mekhlafi *et al*., 2015). In the study conducted through focus group discussion, there has been an outstanding agreement between local disease concepts in the family and society related to diarrhea of 31 mothers, each of them having a single child. The first six cases have also been identified in the region (Senoon, Lafkha, Halib, Didan, Raqaba, and Ayn). Most of these diseases have not been medically treated. Mothers have confidence in traditional medicine and think it is always beneficial and not harmful. The participants do not divulge traditional medicine use to their doctors because doctors face these practices and are not open enough to these kinds of treatment. (Webair and Bin Ghouth, 2014). Socioeconomic status was a predictor in the understanding of prevention, follow-up and treatment of health-related issues, and improvement of public awareness is necessary to decrease morbidity and mortality.

## Conclusion and recommendations

### Conclusion

Through this comprehensive review of studies on children conducted over the last six years of the war, we discovered a shortage in literature covering the aspects relevant to the childhood crisis and serious challenges affecting children’s life and development, widespread epidemics, and an exacerbation of the situation with the second wave of the COVID-19 pandemic currently afflicting the country. Currently published comprehensive analyses are based on the survey conducted eight years ago, making reliability questionable due to considerable changes in demographic quality and quantity. We divided the studies into several categories viz infectious diseases, non-infectious diseases, blood-related diseases, oral and dental problems, accidents and injuries, health system, and family and community issues. The epidemiological situation and research have largely ignored significant issues arisen by the war, especially in the vulnerable areas such as Marib, Al-Jawf, Al-Dhalea, and Saada, as well as injuries and psychological effects on children caused by war. The risks faced by thousands of children who have lost their parents due to murder, capture, and kidnapping also remain largely overlooked. Nevertheless, the child recruitment in war and violence against girls has also been ignored. According to United Nations reports, no studies mention the serious problems related to food security, the denial of education, and the lowest elements of health such as vaccination from deadly diseases that threaten childhood in Yemen and turn Yemen into a focus of epidemics.

### Recommendations

(i) Urgently stop the war, and start an urgent response process to address the devastating effects of war on childhood. (ii) Addressing the current family disintegration situation that the Yemeni families suffer from and reunifying them. (iii) Implementation of a specific response, such as emergency and mobile clinics to save children and distribute nutritional supplements for infants, as well as care for pregnant women and distribution of necessary vaccinations. (vi) Providing psychological and social support services for all children, particularly those exposed to the causes of post-traumatic stress disorder. (vii) Rehabilitating and establishing health units to fulfill the childrenʼs entitlement to adequate medical treatment. (viii) Establishing committees specializing in data collection and supplying schools and health units with instruments for data collection, such as height and weight scales and the malnutrition scale (MOAC), in order to perform current field research based on correct data. (ix) Activating the media aspects in raising awareness, whether for parents of alleviating crises on children or for conflicting parties to respect childhood laws during the war. (x) The concerned authorities and scholars must urgently conduct more field and research studies to accurately assess the childhood crisis.

## Data Availability

Not applicable.
